# Cancer-associated fibroblasts promote oral squamous cell carcinoma progression through LOX-mediated matrix stiffness

**DOI:** 10.1186/s12967-021-03181-x

**Published:** 2021-12-20

**Authors:** Jia-Yi Zhang, Wei-Wen Zhu, Meng-Yao Wang, Run-Dong Zhai, Qiong Wang, Wei-Li Shen, Lai-Kui Liu

**Affiliations:** 1 Department of Basic Science of Stomatology, The Affiliated Stomatological Hospital of Nanjing Medical University, 136# Hanzhong Road, Nanjing, 210029 Jiangsu China; 2 Jiangsu Province Key Laboratory of Oral Diseases, Nanjing Medical University, Nanjing, Jiangsu China; 3 Jiangsu Province Engineering Research Center of Stomatological Translational, Nanjing, Jiangsu China

**Keywords:** Cancer-associated fibroblasts, Epithelial–mesenchymal transition, Lysyl oxidase, Matrix stiffness, Oral squamous cell carcinoma

## Abstract

**Background:**

Cancer-associated fibroblasts (CAFs), the most abundant cells in the tumor microenvironment, have prominent roles in the development of solid tumors as stromal targets. However, the underlying mechanism of CAFs’ function in oral squamous cell carcinoma (OSCC) development remains unclear. Here, we investigated the role of lysyl oxidase (LOX) expression in CAFs in tumor stromal remodeling and the mechanism of its effect on OSCC progression.

**Methods:**

Multiple immunohistochemistry (IHC) staining was performed to detect the correlation of CAFs and LOX in the stroma of OSCC specimens, as well as the correlation with clinicopathological parameters and prognosis. The expression of LOX in CAFs were detected by RT-qPCR and western blot. The effects of LOX in CAFs on the biological characteristics of OSCC cell line were investigated using CCK-8, wound-healing and transwell assay. CAFs were co-cultured with type I collagen in vitro, and collagen contraction test, microstructure observation and rheometer were used to detect the effect of CAFs on remodeling collagen matrix. Then, collagen with different stiffness were established to investigate the effect of matrix stiffness on the progression of OSCC. Moreover, we used focal adhesion kinase (FAK) phosphorylation inhibitors to explored whether the increase in matrix stiffness promote the progression of OSCC through activating FAK phosphorylation pathway.

**Results:**

LOX was colocalized with CAFs in the stroma of OSCC tissues, and its expression was significantly related to the degree of malignant differentiation and poor prognosis in OSCC. LOX was highly expressed in CAFs, and its knockdown impaired the proliferation, migration, invasion and EMT process of OSCC cells. The expression of LOX in CAFs can catalyze collagen crosslinking and increase matrix stiffness. Furthermore, CAFs-derived LOX-mediated increase in collagen stiffness induced morphological changes and promoted invasion and EMT process in OSCC cells by activating FAK phosphorylation pathway.

**Conclusions:**

Our findings suggest that CAFs highly express LOX in the stroma of OSCC and can remodel the matrix collagen microenvironment, and the increase in matrix stiffness mediated by CAFs-derived LOX promotes OSCC development through FAK phosphorylation pathway. Thus, LOX may be a potential target for the early diagnosis and therapeutic treatment of OSCC.

**Supplementary Information:**

The online version contains supplementary material available at 10.1186/s12967-021-03181-x.

## Background

Oral cancer is the sixth most common cancer in the world, and oral squamous cell carcinoma (OSCC) accounts for 90% of oral malignant neoplasms [1, 2]. Although early diagnosis and treatment are developing and improving, the five-year survival rate of patients with OSCC remains low because of distant metastasis and recurrence, and the quality of life of patients has remained a serious problem [3, 4]. Thus, further understanding of the complex pathogenesis of OSCC is needed to develop a more effective therapy.

The tumor microenvironment (TME), a complex meshwork of extracellular matrix (ECM) macromolecules, plays an important role in tumor progression. As a main component of TME, cancer-associated fibroblasts (CAFs) are of great importance for ECM production and interstitial tissue remodeling in the disturbed homeostasis [5], which can induce stiffness and hypoxia in the matrix [6]. Matrix stiffness is one of the most important biomechanical characteristics of TME faced by cancer cells and stromal cells. Recently, there has been a consensus that mechanical stimulation in the TME, such as the alteration of matrix stiffness, can have an impact on the development of solid tumors by a variety of signaling pathways, and then promote invasion and metastasis [7]. Thus, the relationship between CAFs and matrix stiffness and the mechanism of CAFs remodeling ECM are especially worthy of further study.

Collagen is the most common ECM scaffold protein in the matrix [8], and its remodeling and cross-linking critically affect the tumor progression by enhancing ECM stiffness [9]. Previous studies reported that collagen is associated with the development of several malignancies such as breast, colon and liver cancer [7, 10, 11]. Increased collagen deposition and ECM density have been found in the tumor invasion frontier and have been shown to have a significant association with several important clinicopathological parameters [12]. Lysyl oxidase (LOX), a copper-dependent amine oxidase secreted by fibrogenic cells [13], can initiate covalent cross-linking of collagen and elastin in the ECM [14]. The elevation of LOX expression has been found in many malignant tumors [13, 15, 16] and correlates with enhanced cancer cell proliferation, metastasis, and angiogenesis in the TME [17, 18]. LOX can be a molecular target for improving the efficacy of chemotherapies against different types of solid tumors [19], as it has been reported to promote tumor progression by regulating collagen cross-linking [17]. However, the relationship among CAFs, LOX, collagen and their mechanism in the remodeling matrix and influencing tumor progression in OSCC are still largely unknown.

A stiff microenvironment has an effect on many aspects of tumor cells, including internal molecular signaling pathways and cell behaviors [20]. Cellular interactions with the extracellular matrix, especially the state of cell adhesion, have changed in tumor initiation and progression. Focal adhesion kinase (FAK), a prominent signaling molecule in focal adhesions, is over-phosphorylated in multiple cancer progression and is responsible for transmitting extracellular mechanical force to changes in gene expression within cancer cells [14, 21]. The potential of mechanical stimuli such as elevated collagen and tissue stiffness is responsible for the induction of β-catenin translocation and the expression of Wnt-associated proteins [22]. However, the molecular mechanisms driving tissue stiffness-related tumor progression have not been completely elucidated in OSCC.

In the present study, we sought to investigate the correlation between ECM remodeling mediated by CAFs-derived LOX and OSCC development. The colocalization of CAFs and LOX in tumor stroma were explored in OSCC tissues. The expression of LOX in CAFs and its effect on biological behavior of OSCC cells were investigated in vitro. Then, we studied whether LOX expressed by CAFs can significantly catalyze the cross-linking of collagen and increase matrix stiffness, thereby regulating the morphology and invasion of tumor cells in a FAK-dependent manner. Thus, our study suggests that matrix stiffness mediated by CAFs-derived LOX might present a novel therapeutic thought for OSCC patients.

## Methods

### Patients and specimens

Ninety six cases of primary OSCC tissue samples were recruited from 2010 to 2015 from patients treated with surgery at the Department of Oral and Maxillofacial Surgery, the Affiliated Stomatological Hospital of Nanjing Medical University: 59 were male (61.46%) and 37 were female (38.54%), with an average age of 60.4 years (range, 35–91 years). The primary tumors were located in the tongue (n = 32), gingiva (n = 21), buccal mucosa (n = 26), and other sites (n = 17), such as the palate, lower lip, jaw, soft palate, oropharynx, or floor of the mouth. All patients underwent extensive resection of the primary tumor and underwent classic radical neck dissection or selective regional lymph node dissection. None of the patients had received chemotherapy or radiotherapy before the surgery. All patients had complete clinical data and clear pathological diagnosis, including sex, age, tumor location, tumor size, lymph node metastasis, distant metastasis, pathological grade, clinical stage, postoperative recurrence, survival and follow-up information. Clinical stage and TNM classification were defined by the International Union Against Cancer (UICC) and the American Joint Commission on Cancer (AJCC). The pathological classification was based on the World Health Organization (WHO) criteria. This study was approved by the ethics committee of Nanjing Medical University and was conducted in accordance with the World Medical Association Declaration of Helsinki. Written informed consent was obtained from all the patients.

### Multiple immunohistochemical staining

Multiplex IHC staining was performed using Polychromatic fluorescent IHC Kit (CAT#:10080100050, PANOVUE, China). Formalin-fixed, paraffin-embedded primary OSCC tissue specimens were cut into 4 μm-thick sections, cleared with xylene, and rehydrated through a graded alcohol series. After antigen retrieval and blocking, the sections were incubated overnight with primary antibodies against as follows: LOX (1:100; Abcam, Cambridge, MA, USA), α-SMA (1:200; Abcam), EPCAM (1:200; Abcam), FAP (1:250; Abcam). Followed by secondary antibodies, fluorophore (FITC, Cy3, Cy5)-conjugated TSA signal amplification buffer were added. After three sequential reactions, sections were counterstained with DAPI and mounted with enhanced anti-fluorescence quencher. Observation was under laser confocal microscope (Zeiss, Oberkochen, Germany).

For immunohistochemical staining, after incubated overnight with primary antibodies against LOX (1:100; Abcam) and α-SMA (1:200; Abcam), all sections were incubated with HRP-polymer anti-rabbit Kit and a DAB Detection Kit (Maixin Biotech, Fuzhou, China) followed by counterstaining with hematoxylin. The sections were dehydrated in gradient alcohol, clarified in xylene, mounted with neutral gum. Observation was under microscope (Leica Microsystems, Mannheim, Germany).

### Evaluation of immunoreactivity

The staining intensity of tissue sections was individually evaluated by two pathologists who were blinded to the patients' clinical data. Immunoreactivity of LOX and α-SMA in tumor stroma was estimated according to the classification system described by Fuji et al. [23]: 0, negative (no staining); 1, sparse (a small population of discrete stained fibroblasts); 2, focal (irregular and intermittent areas of stained fibroblasts); and 3, abundant (widespread and consecutive areas of stained fibroblasts). The specimens were categorized into two subgroups according to immunoreactivity score: low expression (0–1) and high expression (2–3).

### Fibroblast isolation and preparation of conditioned media

Fresh oral cancer tissues and matched normal tissues were collected under sterile conditions. All samples were obtained with informed consent from the patients, and the fibroblast isolation protocol was approved by the Nanjing Medical University Ethics Committee. All tissue samples were snap-frozen in liquid nitrogen and stored at − 80 °C until fibroblast isolation. Fresh tissues were washed with PBS (penicillin 100 U/mL, streptomycin 100 μg/mL) three times and cut into approximately 1 mm^3^ size. The tissues were spread evenly on the culture dish and placed upside down in a primary incubator for 4 h to adhere. The tissues were maintained in DMEM (Gibco, Grand Island, NY, USA) with 15% FBS (Gibco) for 1–2 weeks. Purified fibroblasts were obtained by differential enzyme digestion and maintained in DMEM with 10% FBS. Cells were seeded into T-25 culture flasks and grown in 5 ml serum-free media for 48 h until the cells were ~ 80% confluent. Conditioned media were collected and centrifuged at 2000 rmp for 30 min to remove cellular debris.

### Cell culture

HNSCC cell lines Cal27 and HN6 were used. Cal27 and HN6 cell lines were purchased from the American Type Culture Collection (ATCC, Manassas, VA, USA). All cancerous cell lines were maintained in Dulbecco’s modified Eagle’s medium (DMEM)/F12 (Invitrogen) supplemented with 10% fetal bovine serum (Gibco) and 1% penicillin/streptomycin at 37 °C in a 5% CO_2_-humidified incubator. All cells were routinely tested for mycoplasma at regular intervals throughout the whole course of the study. To inhibit of FAK phosphorylation, cells were treated with FAK inhibitor 14 (FAKi, Santa Cruz Biotechnology, Santa Cruz, CA, USA) at different concentrations.

### Lentiviral construct and transfection

Human LOX-encoding lentiviral vectors were constructed by GeneChem Co., Ltd (Shanghai, China). CAFs cells were transfected with a specific shRNA lentivirus targeting LOX knockdown, and the scrambled sequence was co-transfected as a negative control. All transfection procedures were performed according to the manufacturer’s instructions. Cells in the control group (CAFs-NC) and the experimental group (CAFs-shLOX) were cultured at 37 °C in a 5% CO_2_ incubator and changed to complete medium 12–16 h after transfection. Stable cells were selected using 2 mg/ml puromycin. Fluorescence microscopy was used to observe transfection efficiency, and real-time polymerase chain reaction (PCR) and western blot were used to estimate the efficiency of LOX knockdown.

### RNA isolation and quantitative reverse transcription-quantitative PCR (qRT-PCR)

Total RNA was extracted from cells with TRIzol reagent (Invitrogen) and equal quantities of cDNA were reverse transcribed using Superscript (Vazyme, Nanjing, China) according to the manufacturer’s instructions. qPCR was performed using the PCR System 7900 (Applied Biosystems, Foster City, CA, USA) with SYBR Green Master Mix (TaKaRa, Shiga, Japan). The primers for GAPDH were: forward 5′-GAAGGTGAAGGTCGGAGTC-3′ and reverse 5′-GAGATGGTGATGGGATTTC-3′; LOX: forward 5′-TTCTTACCCAGCCGACCAAGATA-3′ and reverse 5′-GTGTTGGCATCAAGCAGGTCA-3′; α-SMA: forward 5′-CTGGCCGAGATCTCACTGACTA-3′ and reverse 5′-GCCCATCAGGCAACTCGTAA-3′; FAP: forward 5′-GACCCACGCTCTGAAGACAGAATTA-3′ and reverse 5′-AGCAGAGGTGGCAACTCCAAATAC-3′; FSP-1: forward 5′-CAGATAAGCAGCCCAGGAAGA-3′ and reverse 5′-AAGGAGCCAGGGTGGAAAA-3′; E-cadherin: forward 5′-TACACTGCCCAGGAGCCAGA-3′ and reverse 5′-TGGCACCAGTGTCCGGATTA-3′; N-cadherin: forward 5′-TGGACCATCACTCGGCTTA-3′ and reverse 5′-ACACTGGCAAACCTTCAC-3′; vimentin: forward 5′-TGAGTACCGGAGACAGGTGCAG-3′ and reverse 5′-TAGCAGCTTCAACGGCAAAGTTC-3′. The following program was performed using a two-step cycling protocol: an initial denaturation at 95 °C for 30 s, followed by 40 cycles of 95 °C for 5 s, 60 °C for 31 s. Each sample in each experiment was performed in triplicate and the results were expressed as the mean of three independent experiments. Relative expression levels of related genes were quantified and compared to the internal control GAPDH and analyzed using the 2−ΔΔCT method.

### Protein extraction and nuclear, cytoplasmic protein fractionation

Protein extraction was performed using common methods. Briefly, cells were lysed with an appropriate radioimmunoprecipitation assay (RIPA) buffer (Beyotime, Shanghai, China) containing protease inhibitor cocktail and phosphatase inhibitor cocktail (Beyotime, Shanghai, China), incubated for 15 min at 4 °C, and scraped using a cell scraper. For cells cultured on collagen, cells were agitated on a rocking platform at 4 °C for 30 min and then removed the lysis buffer. Nuclear and cytoplasmic proteins were isolated using the Invent Nuclear and Cytoplasmic Extraction Reagents Kit (Invent Biotechnologies, Plymouth, MN, USA) according to the manufacturer’s instructions.

### Western blot assay

Western blot assays were performed according to the common methods. Briefly, equal amounts of protein samples were separated using sodium dodecyl sulfate polyacrylamide gel electrophoresis (SDS-PAGE) and transferred to polyvinylidene fluoride (PVDF) membranes (Millipore, Billerica, MA, USA). The membranes were blocked with 5% skimmed milk at room temperature for 2 h and incubated at 4 °C overnight with primary antibodies against β-actin as a control (1:500; Proteintech), α-SMA (1:200; Abcam), FSP-1 (1:1000; Abcam), FAP (1:800; Abcam), LOX (1:1000; Abcam), E-cadherin (1:1000; CST), N-cadherin (1:1000; CST), vimentin (1:500; Santa Cruz Biotechnology), β-catenin (1:1000; CST), FAK (1:1000, CST), p-FAK(Tyr397) (1:1000, CST), c-myc (1:500, CST), cyclin D1 (1:500, CST), Lamin B1 (1:1000, CST) followed by incubation with anti-goat immunoglobulin G (IgG) horseradish peroxidase (HRP)-conjugated secondary antibodies (Zhongshan Golden Bridge Bio, Beijing, China) for 45 min at room temperature. The protein bands were detected using ECL chemiluminescence reagents (Millipore) and visualized using the ImageQuant LAS4000 Mini Imaging System (General Electrics, Louisville, Kentucky). Analyses of the bands were performed using ImageJ software.

### Cell proliferation assay

Cell proliferation was assessed by CCK-8 cell assay (Cell Counting Kit-8, Dojindo, Japan). Cells (3 × 10^3^ cells/well) were seeded into 96-well plates, and 10 μl CCK-8 reagent was added to each well 2–4 h before testing at the same time. Cell viability was determined at 0, 1, 2, 3 and 4 days after cell attachment. Three parallel repeats were performed for each sample in each experiment and the results were expressed as the mean of three independent experiments.

### Cell migration and invasion assays

Cell migration and invasion assays in vitro were performed using wound healing and transwell assays. For the wound healing assay, cells were plated in six-well plates and grown to 90% confluence. Artificial wounds were created with a 200 μl sterile pipette tip across the cell surface and then the cells were incubated with CM to migrate into the open area. Images of the same area of the wound were taken at 0, 12 and 24 h to determine the wound closure. Cell invasion assays were performed using 8 μm pore size transwell chambers (Miliipore) whose upper chamber was precoated with Matrigel (Corning, Bedford, MA, USA). For the migration assays, there was no matrigel in the upper chamber. Approximately 1 × 10^5^ cells per well were seeded in the upper chambers and incubated with CM in the lower chamber. After 24 h, the transwell chambers were fixed with 4% PFA and stained with crystal violet (Sigma-Aldrich, St. Louis, MO, USA). Cells attached to the lower layer were imaged (Olympus, Tokyo, Japan) and counted in randomly selected fields.

### Preparation of collagen matrix

For the fibroblast-modified collagen, fibroblasts were resuspended in FBS, and type I collagen, 5 × DMEM and reconstitution buffer (50 mmol/L NaOH, 260 mmol/L NaHCO_3_, and 200 mmol/L HEPES) were sequentially added and evenly mixed. The mixture was spread in a 12-well plate, and the plate was placed in a 5% CO_2_ incubator at 37 °C for 30–60 min to solidify. Complete medium was added and the medium was changed every day. For the collagen treated with CAFs-CM, CM from CAFs-NC, CAFs-shLOX were collected to stimulate the formation of collagen with different stiffness. DMEM was used as blank control, and human recombinant LOX (huLOX; OriGene, Rockville, MD, USA) was used for the rescue of LOX knockdown. 12-well plates were coated with 1.5 mg/ml collagen and placed at room temperature for 30 min followed by incubation at 37 °C for 2 h, and then stimulated by DMEM, CM from CAFs-NC, CAFs-shLOX and CAFs-shLOX + huLOX for 5 days. For the collagen treated with drugs, huLOX was used to induce crosslinking of collagen. Ribose (Sigma-Aldrich, Dorset, UK), which induced crosslinking non-enzymatically, was used as a positive control and BAPN (Sigma-Aldrich, Dorset, UK) was used as an inhibitor of LOX. Then, PBS only (PBS), or PBS containing 150 ng/ml huLOX (huLOX), or 60 mM ribose (ribose), or huLOX combined with 10 mM BAPN (huLOX + BAPN) were added to each group and incubated at 37 °C for 5 days.

### Cellular immunofluorescence assay

For cellular immunofluorescence staining, cells were attached to cell slides in 24-well plates. For F-actin staining, the cells were grown on collagen gels for 24 h. Then the cell slides and collagen were fixed in 4% PFA for 20 min at room temperature and permeabilized with 0.1% Triton X-100 for 15 min. Then incubated with primary antibody against E-cadherin (1:1000; CST), vimentin (1:500; Santa Cruz Biotechnology), F-actin (1:100, Beyotime) overnight at 4 °C. Nucleus were stained with 4,6-diamidino-2-phenylindole (DAPI) (Beyotime, Shanghai, China). Fluorescence images were visualized using a fluorescence microscope (Leica Microsystems, Mannheim, Germany).

### Determination of matrix stiffness

The relative stiffness of collagen gels was measured using a MARS60 microinfrared rheometer (Thermo Fisher, MA, USA) under the following conditions. Briefly, the collagen gels were measured over a range from 0.5–5% strain at a fixed angular frequency of 0.5 rad/s and temperature of 21 °C on MARS60 microinfrared rheometer. The output of G’ was recorded for 20 min with a data point obtained every 60 s. The samples were found to be only minimally frequency dependent within the range of testing and showed a linear viscoelastic response within the strain range evaluated.

### Three-dimensional co-culture system

Fibroblast-modified collagen was prepared as previously described. Cal27 cells were resuspended in the mixed medium and then seeded onto the surface of the solidified gel to create a 3D co-culture system. The culture medium was changed daily. After 3 days, the collagen gels were transferred to the scaffold in six-well plates for culture at the air–liquid interface. At the specific time point, the gels were fixed with 4% PFA, embedded in paraffin, and cut into 4 μm sections for H&E staining.

### Statistical analysis

The correlation of α-SMA and LOX expression with clinicopathological parameters and prognosis was analyzed using ANOVA, the chi-square test, Fisher’s exact test, Mann–Whitney test, COX regression analysis and Kaplan‐Meier survival analysis. Spearman's correlation analysis was used to estimate the correlation between α-SMA and LOX in the tumor stroma. The results are represented as the means ± SEM or as specified in the figure legends. The data were analyzed using SPSS 18.0 and Prism 7 software (GraphPad Software, La Jolla, CA, USA). Comparisons between the two groups were analyzed using the 2-tailed, unpaired Student’s t test. Comparisons among the three groups were performed using one-way ANOVA followed by Tukey’s multiple comparisons test. Adjusted values of P < 0.05 were considered significant.

## Results

### Expression of LOX in the stroma of OSCC is associated with CAFs

**Fig. 1:**
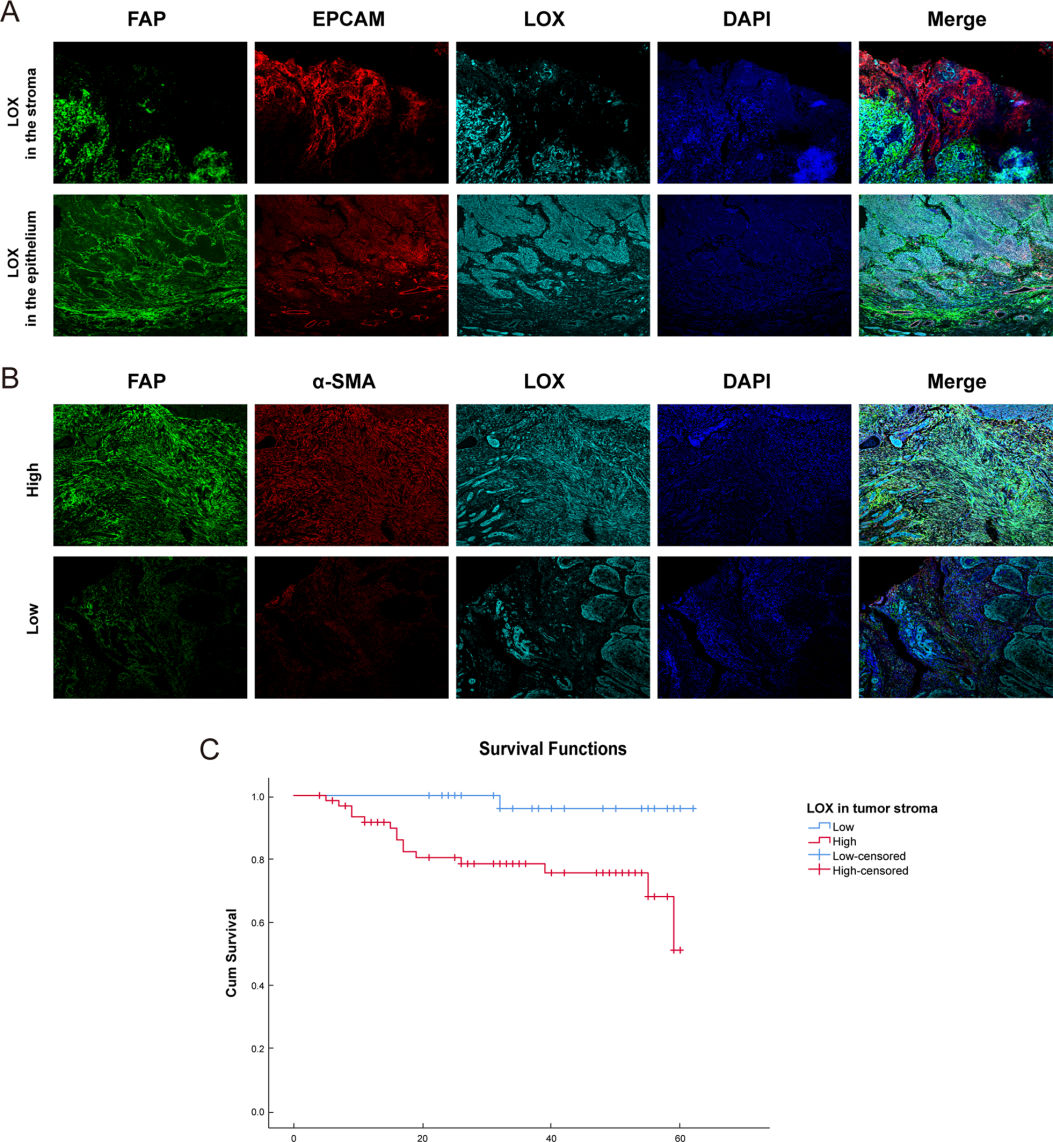
LOX expressed in tumor stroma is colocalized with CAFs. **A** Distribution of LOX expression was detected by multiple IHC staining. Representative images at 100 × magnification. FAP (green), EPCAM (red), LOX (cyan), DAPI (blue). **B** Colocalization of LOX and CAFs in the stroma were shown. Representative images of high and low expression levels of FAP, α-SMA and LOX in tumor stroma at 100 × magnification. FAP (green), α-SMA (red), LOX (cyan), DAPI (blue). **C** The Kaplan–Meier survival curves of prognostic models based on LOX expression in tumor stroma for patients with OSCC

The fibroblast marker FAP, the epithelial marker EPCAM, and LOX were stained by multiplex immunohistochemical (IHC) staining to evaluate the expression distribution of LOX in OSCC tissues. We found that LOX was highly expressed both in the epithelium and stroma of OSCC (Fig. 1A). To investigate the expression of LOX in tumor stroma, we also evaluated the expression of LOX and CAFs markers α-SMA and FAP in OSCC clinical samples by multiplex IHC staining. The results showed that CAFs were located in the tumor stroma by staining α-SMA and FAP. Moreover, the high expression of LOX in tumor stroma was observed in the same regions of the high expression of α-SMA and FAP. LOX was not expressed or expressed at low levels in the region with the low expression of α-SMA and FAP, suggesting the colocalization between LOX and CAFs (Fig. 1B).

Then, the sections of 96 OSCC clinical samples were subjected to IHC staining to evaluate the expression of α-SMA and LOX in the stroma. α-SMA and LOX were separately stained in serial sections of OSCC samples. The results showed that in 96 cases of OSCC, 56.25% (54/96) exhibited relatively high expression of α-SMA (Additional file 1: Fig. S1A1-3), and 43.75% (42/96) exhibited low expression (Additional file 1: Fig. S1C1-3). Meanwhile, 64.58% (62/96) showed the high expression of LOX (Additional file 1: Fig. S1B1-3), whereas the remaining 35.42% (34/96) showed the low expression (Additional file 1: Fig. S1D1-3). The association between these two parameters was analyzed, as shown in Table 1, LOX expression in the stroma of OSCC was positively associated with α-SMA expression (*P* = 0.008), further verifying the correlation of the two.

**Table 1:** The relationships between α-SMA and LOX expression in tumor stroma in OSCC

Variable	No.	LOX expression		
		Low	High	P
α-SMA expression				**0.008**
Low	42	21	21	
High	54	13	41	

No. Number of patients; Bold values signify P < 0.05.

### Association of clinicopathological parameters with α-SMA and LOX expression in the stroma of OSCC

The staining results of 96 OSCC clinical samples were scored based on the IHC staining scoring standards, and their relationship with the clinicopathological parameters was evaluated. Notably, α-SMA expression in CAFs significantly correlated with tumor size (Table 2, *P* = 0.028), and the higher expression of α-SMA was observed in patients with advanced clinical stages (Table 2, *P* = 0.012). In addition, LOX expression in the stroma of OSCC was significantly associated with the advanced clinical stage (Table 2, *P* = 0.042). The correlations between α-SMA or LOX expression with patient age, sex, tumor location, lymph node metastasis, distant metastasis and pathological grade could not be identified.

**Table 2:** Associations between clinical variables and expression levels of α-SMA and LOX in stroma

Variable	No.	α-SMA expression			LOX expression		
		Low	High	*P-*value	Low	High	*P-*value
Sex				0.731			0.356
Male	59	25	34		23	36	
Female	37	17	20		11	26	
Age(years)				0.854			0.54
≤ 50	22	10	12		9	13	
> 50	74	32	42		25	49	
Tumor location				0.308			0.662
Tongue	32	14	18		11	21	
Gingiva	21	6	15		9	12	
Buccal mucosa	26	12	14		7	19	
Others	17	10	7		7	10	
Tumor size				**0.028**			0.212
T1	18	12	6		9	9	
T2	43	18	25		14	29	
T3	22	9	13		8	14	
T4	13	3	10		3	10	
Lymph node metastasis				0.496			0.527
N0	58	27	31		22	36	
N	38	15	23		12	26	
Distant metastasis							
M0	96	42	54		34	62	
M	0	0	0		0	0	
Clinical stage				**0.012**			**0.042**
I	13	10	3		7	6	
II	25	11	14		11	14	
III	30	13	17		9	21	
IV	28	8	20		7	21	
Pathological grade				0.986			0.208
I	58	25	33		23	35	
II	30	14	16		9	21	
III	6	2	4		1	5	

No., number of patients; N0, no lymph node metastasis; N, lymph node metastasis; M0, no metastasis; M, metastasis; Bold values signify P < 0.05.

Additionally, the COX proportional hazards regression model and the Kaplan‐Meier survival analysis were used to further investigate the prognostic significance of LOX and α-SMA expression in the stroma of OSCC. As shown in Tables 3, 4, the univariate and multivariate COX analyses indicated that LOX expression in tumor stroma could serve as an independent prognostic factor for the overall survival (OS) of OSCC patients. The Kaplan‐Meier survival analysis showed that higher expression of LOX in tumor stroma had an adverse prognostic impact on the OS of OSCC patients (Fig. 1C, P < 0.001). Based on these results, it was concluded that the stromal expression of LOX was significantly associated with the malignant grade and poor prognosis of OSCC.

**Table 3:** Univariate COX regression analysis of overall survival

Variables	P-value	Risk ratio	95% CI
Sex (male, female)	0.464	1.444	(0.54, 3.862)
Age (≤ 50, > 50)	0.805	0.995	(0.957, 1.035)
Tumor location (tongue, gingiva, buccal mucosa, palate, lower lip, jaw, others)	0.803	0.964	(0.725, 1.283)
Tumor size (T1–T4)	0.63	1.128	(0.69, 1.844)
Lymph node metastasis	0.977	1.01	(0.522, 1.954)
Clinical stage (I, II, III, IV)	0.633	1.122	(0.7, 1.798)
Pathological grade	0.96	0.98	(0.434, 2.213)
Local infiltration	0.816	0.881	(0.303, 2.562)
α-SMA in tumor stroma (low, high)	0.928	0.955	(0.35, 2.603)
**LOX in tumor stroma ( low, high)**	**0.025**	**10.088**	(**1.332, 76.419)**

*CI* confidence interval

Bold values signify P-value < 0.05.

**Table 4:** Multivariate COX regression analysis of overall survival

Variables	P-value	Risk ratio	95% CI
Sex (male, female)	0.841	1.117	(0.378, 3.299)
Age (≤ 50, > 50)	0.795	0.995	(0.954, 1.037)
Tumor location (tongue, gingiva, buccal mucosa, palate, lower lip, jaw, others)	0.906	1.02	(0.737, 1.41)
Tumor size (T1–T4)	0.656	1.231	(0.492, 3.081)
Lymph node metastasiss	0.894	1.089	(0.309, 3.845)
Clinical stage (I, II, III, IV)	0.762	0.834	(0.256, 2.712)
Pathological grade	0.516	0.72	(0.267, 1.94)
Local infiltration	0.676	1.313	(0.367, 4.702)
α-SMA in tumor stroma (low, high)	0.62	0.735	(0.217, 2.487)
**LOX in tumor stroma ( low, high)**	**0.017**	**13.062**	**(1.58, 107.992)**

*CI* confidence interval

Bold values signify P-value < 0.05

### LOX-positive CAFs enhance proliferation, migration, invasion and EMT process in OSCC cells

**Fig. 2:**
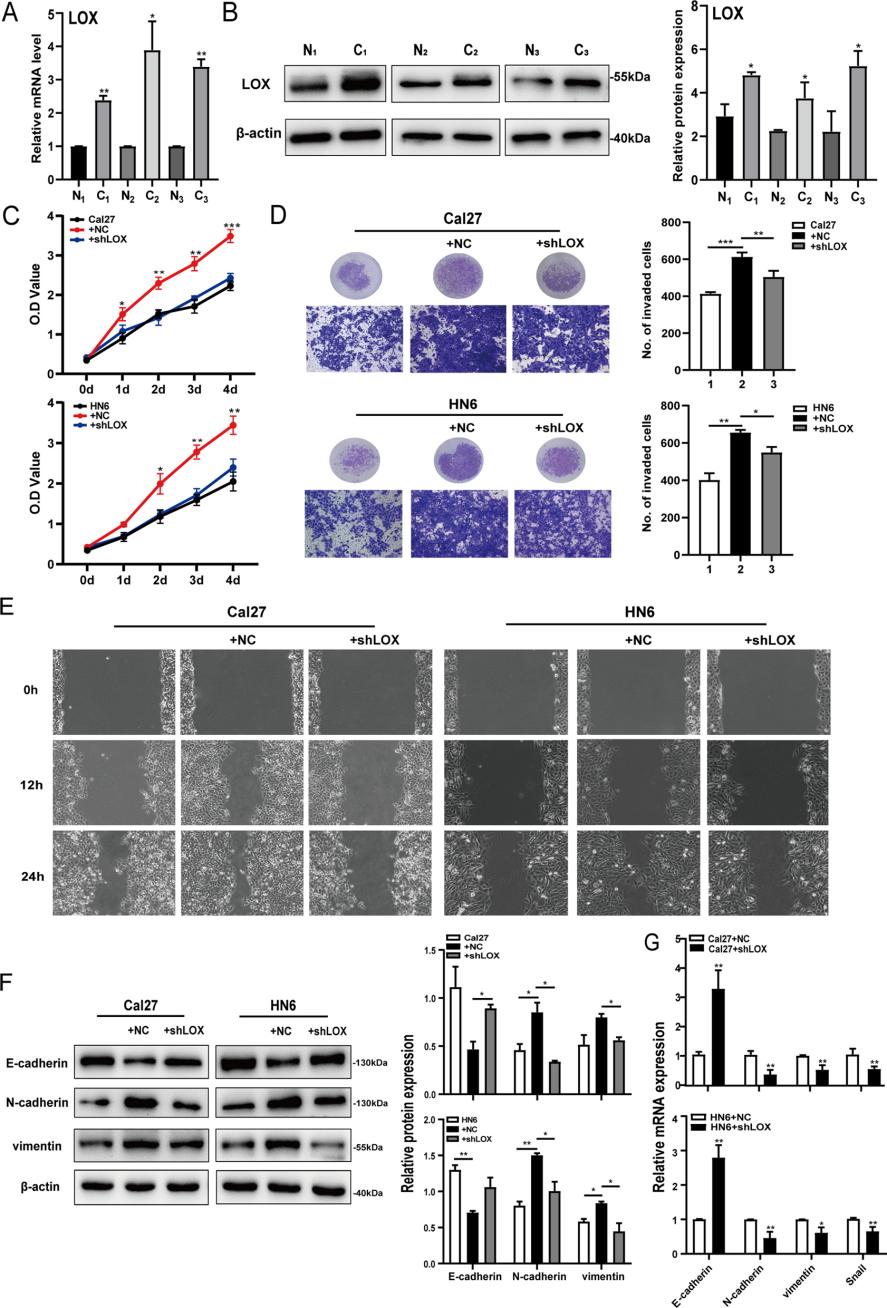
LOX expressed by CAFs affects the biological characteristics of OSCC cells. **A** Gene expression of LOX in three paired NFs and CAFs measured by RT-qPCR. **B** Protein levels of LOX in three paired NFs and CAFs determined by western blot. **C** CCK-8 assay was used to evaluate the proliferation ability of Cal27 and HN6 cells. **D** The invasion ability in Cal27 and HN6 cells were evaluated by transwell assay. Representative images of invaded cells and quantification data were shown. **E** The migration ability in Cal27 and HN6 cells were detected by the scratch test. Representative images were shown. **F** Expression of EMT markers in Cal27 and HN6 cells were examined by western blot. **G** Quantitation of EMT markers mRNA levels were determined by RT-qPCR. GAPDH served as loading control; β-actin served as loading control. The data are presented as the means ± SD (n = 3); *P < 0.05, **P < 0.01, ***P < 0.001

Primary CAFs and normal fibroblasts (NFs) were obtained from fresh human oral cancer tissues and paired adjacent non-tumor tissues. The morphology of purified NFs and CAFs were shown in Additional file 2: Fig. S2A. The expression of CAFs classical markers in three paired tissues were verified by RT-PCR and western blot (Additional file 2: Fig. S2B, C). The study found that LOX was highly expressed in CAFs compared with matched NFs (Fig. 2A, B), which was in line with the clinical results and proved the correlation between LOX and CAFs. To characterize the biological roles of LOX-positive CAFs in OSCC, a loss-of-function assay was performed by knocking down LOX expression through lentivirus transfection. As shown in Additional file 2: Fig. S2D, E, the protein and RNA expression levels of LOX significantly decreased in CAFs transfected with shRNA lentivirus (CAFs-shLOX), compared with that in the control group (CAFs-NC). Then, CAFs-NC and CAFs-shLOX conditioned medium were harvested to stimulate oral cancer cells Cal27 and HN6. The CCK-8 assay showed that the downregulation of LOX in CAFs inhibited the proliferation and viability of Cal27 and HN6 cells (Fig. 2C). Meanwhile, after LOX knockdown in CAFs, the invasive ability of both cell lines was significantly reduced as measured by the transwell assay (Fig. 2D). The scratch test and the migration assay showed that the migratory ability of oral cancer cells was weakened upon LOX knockdown (Fig. 2E; Additional file 2: Fig. S2F). Changes in the expression of EMT markers were also related to tumor progression in vitro. As shown in Fig. 2F, G, the expression of epithelial marker E-cadherin was upregulated in OSCC cells upon LOX suppression in CAFs, while the expression of mesenchymal markers vimentin and N-cadherin were downregulated. To further demonstrate the occurrence of EMT in OSCC cells, we performed immunofluorescence assay and found it consistent with the above-mentioned results (Additional file 2: Fig. S2G). These results confirmed that LOX expression in CAFs promoted the proliferation, migration, invasion and EMT of OSCC cells.

### Effect of LOX-positive CAFs on matrix collagen stiffness

**Fig. 3:**
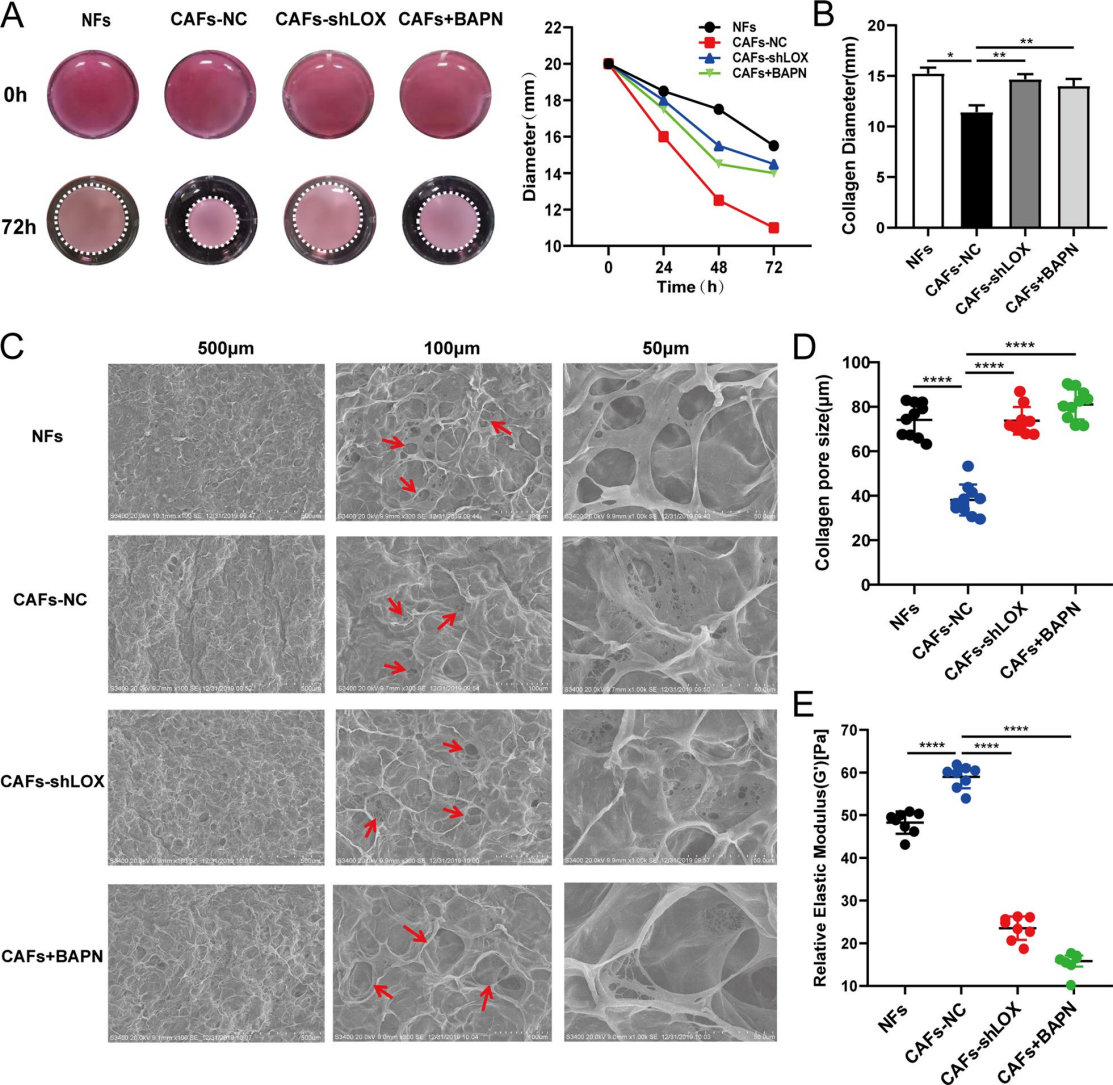
LOX expressed by CAFs affects ECM remodeling in vitro. **A** NFs and CAFs were co-cultured with collagen gels and the contraction of collagen gels was assessed photographically at 0 h and 72 h. Collagen gels diameter was measured every day. **B** The diameter of collagen in each group was statistically analyzed. **C** Representative microscope images of collagen co-cultured with NFs and CAFs were taken by SEM. **D** Quantitative analysis of collagen pore size was calculated by ImageJ. **E** Relative stiffness of collagen were measured by MARS60 microinfrared rheometer. Data was representative of three independent experiments. *P < 0.05, **P < 0.01, ***P < 0.001, ****P < 0.0001

To investigate the effect of LOX-positive CAFs on matrix collagen stiffness, type I collagen was used for co-cultured with NFs and CAFs cells. The contractility of collagen in the CAFs-treated group was significantly stronger than that in the NFs-treated group. However, the contractile capacity of collagen was reduced when LOX was knocked down by lentivirus or inhibited by BAPN in CAFs (Fig. 3A). The changes in collagen diameter in different groups were consistent with its contractility (Fig. 3B). Meanwhile, the pore size of collagen in the CAFs group was significantly smaller than that in the NFs group, whereas the downregulation of LOX reversed this effect (Fig. 3C, D). These results indicated that LOX expression in CAFs could directly promote the crosslinking of collagen, resulting in an increase in contractile capacity and a reduction in the pore size of collagen. To detect collagen stiffness, a MARS60 microinfrared rheometer was used to evaluate the elastic modulus of collagen. The elastic modulus of collagen in the CAFs group significantly increased compared with that in the NFs group, while the elastic modulus reduced upon LOX inhibition (Fig. 3E). Collectively, these data confirmed that LOX expressed in CAFs played an important role in ECM remodeling, leading to further changes in matrix collagen stiffness.

### Effect of matrix stiffness on the biological behavior of OSCC cells

**Fig. 4:**
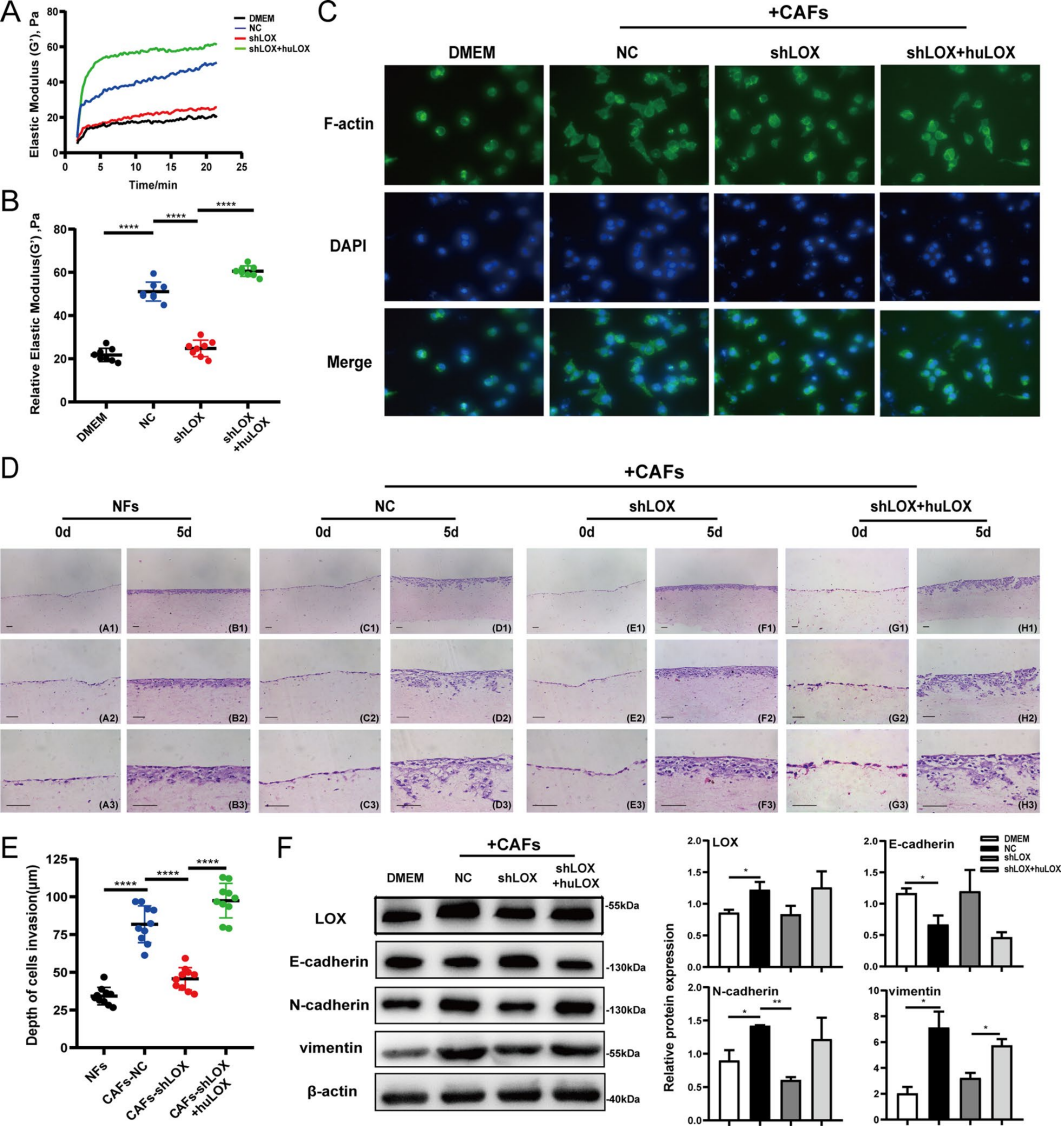
Matrix stiffness mediated by CAFs-derived LOX promotes invasion ability of OSCC cells. **A** Elastic modulus(G’) of type I collagen stimulated by CM from CAFs cells appeared to change over time. **B** Relative stiffness of collagen in each groups under stable condition was shown. Elastic modulus(G’) were measured by MARS60 microinfrared rheometer. **C** Representative immunofluorescence images of F-actin staining in Cal27 cells were shown. Green: F-actin; Blue: DAPI (400 ×). **D** Representative H&E staining images of Cal27 cells cultured with NFs and CAFs in 3D co-culture system were shown. (A1-H1, 100 × ; A2-H2, 200 × ; A3-H3, 400 ×). **E** The depth of Cal27 cells invasion into the collagen was calculated using imageJ (n = 5 per condition). **F** The protein expression of LOX and EMT markers in Cal27 cells cultured on collagen gels were measured by western blot assays. β-actin served as loading control. The data are presented as the means ± SD; Scale bar: 50 μm; *P < 0.05, **P < 0.01, ***P < 0.001, ****P < 0.0001

The interaction between cancer cells and the microenvironment can affect tumor growth and progression [7]. To explore the effects of LOX-mediated matrix stiffness on the biological behavior of oral cancer cells, CM extracted from CAFs-NC and CAFs-shLOX cells was added to type I collagen, on which oral cancer cells were cultured. The relative stiffness of stimulated collagen was measured by rheology. The results showed that collagen in each group reached a steady state in ~ 15–20 min (Fig. 4A), indicating that collagen substrates with different stiffness were successfully constructed. CM from CAFs-NC led to an increase in the stiffness of collagen, which was inhibited by CM from CAFs-shLOX, and this inhibition could be rescued by huLOX (Fig. 4B). Similarly, type I collagen was modified by huLOX, ribose or BAPN to construct cell culture substrate, and the relative elastic modulus was measured (Additional file 3: Fig. S3A), in order to further verify the effect of matrix stiffness on the progression of OSCC.

Cell morphology is related to cell motility, and abnormal changes are important factors influencing cell biological behavior. Therefore, Cal27 cells were seeded onto collagen with different stiffness. Immunofluorescence staining for cytoskeletal protein F-actin showed that Cal27 cells became slender in the CAFs-NC group compared with the control group, and returned to normal morphology in the CAFs-shLOX group (Fig. 4C), suggesting that matrix stiffness mediated by CAFs-derived LOX had a certain effect on the morphological changes in oral cancer cells. Furthermore, the 3D collagen co-culture system showed that the number and depth of tumor cells invading into the collagen substrate were higher in the CAFs-NC group than in the NFs group, which were reduced by LOX knockdown in CAFs (Fig. 4D, E), proving the effect of matrix stiffness on the invasion ability of tumor cells. Meanwhile, rescue experiment were carried out for further confirmation, and we found that huLOX can reversed the morphological change and invasion ability of Cal27 cells in the CAFs-shLOX group. Also, immunofluorescence staining and transwell assays with drug-modified collagen showed that LOX-mediated high collagen stiffness led to deformation and promoted cancer cell invasion (Additional file 3: Fig. S3B, C). Furthermore, western blot assay showed that the increase in matrix stiffness might induce LOX expression, promote N-cadherin and vimentin expression, and reduce E-cadherin expression, suggesting that the increase in matrix stiffness promoted EMT in OSCC cells (Fig. 4F; Additional file 3: Fig. S3D). Collectively, these results suggested that CAFs-derived LOX-mediated changes in the biomechanical properties (stiffness) of the cellular environment directly affected the cellular behavior.

### Matrix stiffness promotes OSCC progression via FAK signaling

**Fig. 5:**
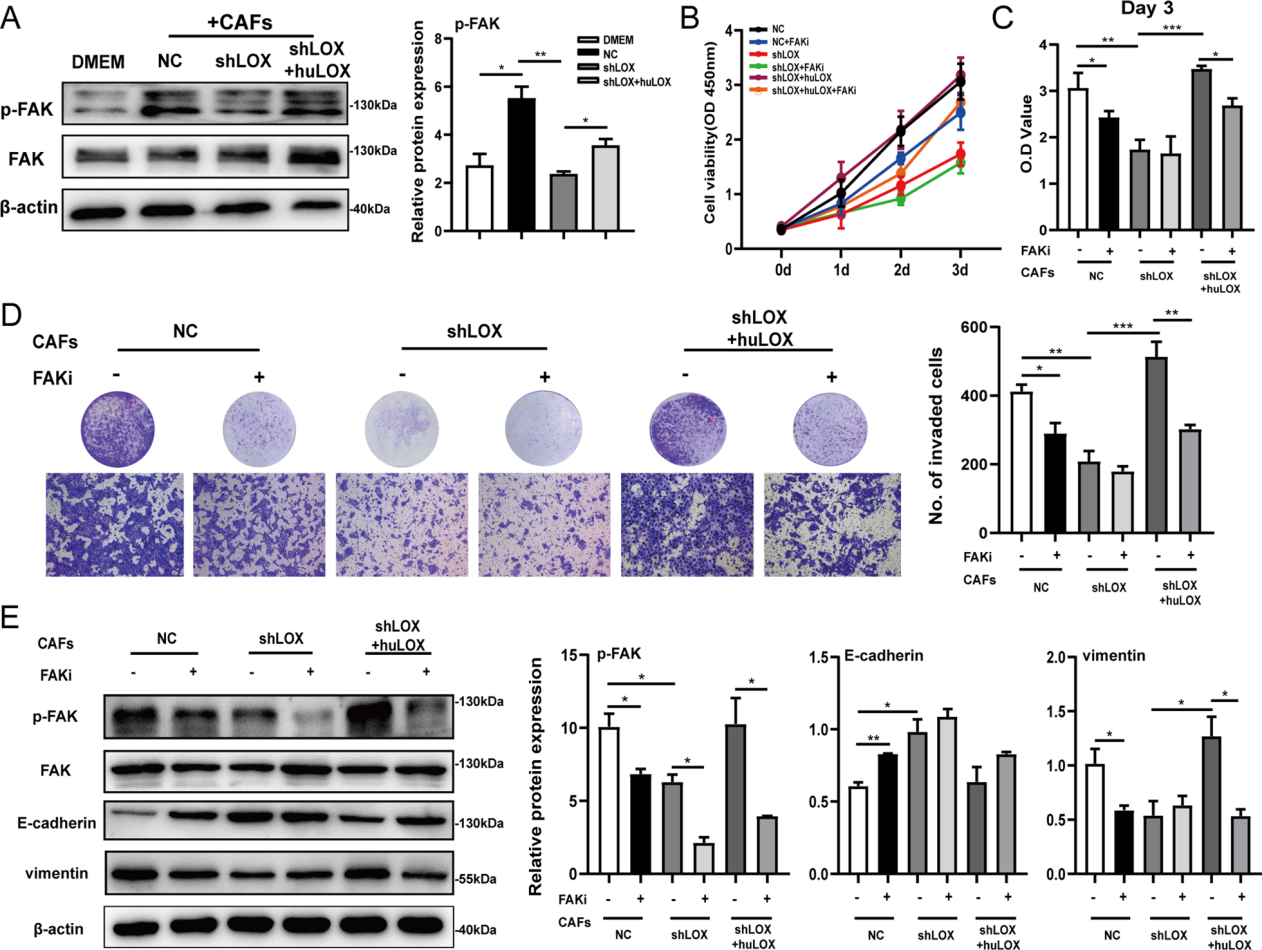
Matrix stiffness mediated by CAFs-derived LOX promotes OSCC progression via FAK signaling pathway. **A** The protein expression of p-FAK and FAK were measured by western blot assay in Cal27 cells cultured on collagen gels. **B**, **C**, The proliferation ability of Cal27 cells on collagen gels after FAKi treatment was measured by CCk-8 assay. **D** The invasion ability of Cal27 cells after FAKi treatment was measured via transwell chambers coated with collagen gels. Representative images and quantification data were shown. **E** The protein expression of p-FAK, FAK and EMT-related markers were measured by western blot assay in Cal27 cells after FAKi treatment. β-actin served as loading control. *P < 0.05, **P < 0.01, ***P < 0.001, ****P < 0.0001

Considering the critical role of FAK in regulating the biological behaviors of tumor cells, such as adhesion, invasion and EMT [21, 24], we speculated that matrix stiffness might promote tumor progression by activating the FAK signaling pathway. Thus, the FAK phosphorylation level at tyrosine 397 was detected in OSCC cells cultured on collagen with different stiffness. The western blot assay showed that the FAK phosphorylation was upregulated in the CAFs-NC group and downregulated in the CAFs-shLOX group (Fig. 5A). Meanwhile, the same activation of FAK phosphorylation was found on the drug-modified collagen (Additional file 4: Fig. S4A). FAK inhibitor 14 (FAKi), a selective small-molecule inhibitor, could repress the phosphorylation of FAK at tyrosine 397. Here, FAK phosphorylation was suppressed after treatment with FAKi, but the total protein level of FAK showed no difference (Additional file 4: Fig. S4B). The increase in matrix stiffness caused by CAFs-derived LOX promoted the proliferation and invasion ability of OSCC cells, which could be reversed by FAKi (Fig. 5B–D). Furthermore, FAKi inhibited the matrix stiffness-induced EMT process in OSCC cells (Fig. 5E). Meanwhile, we found that the expression of β-catenin and its downstream proteins c-myc and cyclin D1 was increased in the group with the high stiffness of collagen, indicating that the Wnt/β-catenin signaling pathway might be activated (Additional file 4: Fig. S4A). The cell fraction assay showed that the increase in β-catenin was observed both in the cytoplasm and nucleus of OSCC cells cultured on collagen with high stiffness, while FAKi restrained matrix stiffness-stimulated nuclear β-catenin enrichment (Additional file 4: Fig. S4C). These results indicated that the matrix stiffness increased by CAFs-derived LOX induced an increase in the FAK phosphorylation level, which might impact tumor development. Moreover, the activation of the FAK phosphorylation pathway might further promote the nuclear accumulation of β-catenin. Further investigation should be performed to explore the underlying mechanism leading to this change in the expression of β-catenin in the cytoplasm and nucleus mediated by matrix stiffness.

## Discussion

Recently, it has become clear that TME promotes tumor progression [25]. The components and mechanical properties of ECM have aroused concern as important determinants of cancer cell behavior and disease progression. As the major TME cellular component, CAFs play a key role in matrix remodeling [6]. However, whether LOX participates in extracellular matrix remodeling induced by CAFs and affects the progression of OSCC remains unclear. Our study revealed that LOX is highly expressed in CAFs, and LOX can mediate CAFs-induced matrix remodeling by catalyzing collagen cross-linking. The increase in matrix stiffness can, in turn, promote tumor invasion and progression in an FAK-dependent manner.

CAFs are responsible for the secretion of multiple growth factors, kinases, cytokines and chemokines into the TME to facilitate tumor progression. Research identified high expression of LOX in several malignant tumors [26]. The secretion of LOX was reported to induce the formation of a pre-metastatic niche in preclinical breast cancer models [27]. LOX has a significant effect on ECM remodeling in the lungs and promotes lung metastasis [28] and correlates with increased staging in renal cell carcinoma [13]. In previous studies, the expression of LOX was usually stronger at the tumor invasive front than in the tumor center in OSCC, and higher expression of LOX was associated with advanced pathological grade [12]. Here, we found that LOX was highly expressed in the same region of CAFs distribution in tumor stroma (Fig. 1), and there was a statistical correlation between the two (Table 1). Notably, higher expression of LOX in the tumor stroma was associated with an advanced clinical stage and poor prognosis (Tables 2, 3, 4). Moreover, we found that the expression level of LOX was much higher in CAFs than in NFs, and further confirmed that LOX expressed in CAFs functioned as an oncogene in OSCC (Fig. 2). These results suggest that the expression of LOX in tumor stroma may be a key molecule affecting the progression of OSCC.

There has been extensive evidence to prove that biophysical factors, including matrix stiffness and mechanical forces, play critical roles in the initiation and progression of malignant tumors [29–31]. CAFs can produce large quantities of ECM molecules to remodel stroma, and LOX expression and collagen content have been observed to be clinically elevated in various malignant tumors [13, 32–35]. In Kurie’s review, the deposition of a cross-linked collagen matrix by CAFs leads to intratumoral fibrosis [36]. Xu et al. summarized the most abundant matrix protein polymers are collagens, whose crosslinking by LOX can create a stiffer tumor microenvironment, regulate tumor immunity, and promote metastasis [37]. In present study, in order to investigate the effect of LOX in CAFs on matrix remodeling, we created a co-culture system and found that the contractile capacity of collagen was enhanced by CAFs, leading to an increase in collagen density and a decrease in pore size. Meanwhile, the elastic modulus of collagen in the CAFs-co-cultured group was elevated (Fig. 3). Thus, we innovatively explored the relationship between CAFs and LOX, finding that LOX may be a key factor in CAFs to remodel ECM through collagen crosslinking, and then promote cancer progression.

In the development of malignant tumors, the complexity of the epithelial–stromal interaction is due to the fact that the ECM is a dynamic mechanical microenvironment. ECM remodeling leads to functional changes in the biomechanical properties of the ECM, resulting in the activation of pathogenic signaling pathways involved in various processes such as cell morphology, proliferation, differentiation, migration and etc. [30, 38, 39]. In breast carcinoma, mammary epithelial cell cultured within high-density collagen form larger, less organized structures and proliferate faster than that those cultured in low-density collagen [40]. LOX has been identified as playing a critical role in establishing and mediating a prometastatic microenvironment within fibrotic tissues [35]. In the present study, type I collagen was stimulated with CM from CAFs or drugs to form different stiffness, and the morphology of oral cancer cells cultured on high-stiffness collagen became slender. Moreover, a 3D collagen co-culture model was successfully established, in which oral cancer cells were plated on collagen with different stiffness mediated by CAFs. Our results showed that an increase in collagen stiffness could promote the depth of tumor cell invasion into the collagen matrix (Fig. 4). Such changes in the mechanical properties of the ECM arising from LOX-mediated collagen cross-linking play a critical role in the transformed phenotype of oral cancer cells.

Tumors respond to changes in ECM stiffness by integrin activation and subsequent phosphorylation of FAK, leading to the activation of protein–protein interactions and adhesion signaling to promote tumorigenesis [41, 42]. FAK is required for the activation of the Wnt/β-catenin pathway through phosphorylation of GSK3β_Y216_, which is crucial for intestinal tumorigenesis [43]. In Xue’s study, LOXL2 secreted by CAFs activated the FAK signaling pathway and then induced EMT in human colon cancer LOVO cells, thereby promoting the invasion and metastasis of colon cancer (44). By seeding OSCC cells on the collagen with different stiffness, we found that collagen with a stiffer surface mediated by CAFs-derived LOX can trigger the EMT process, which was consistent with cell morphological changes. A stiffer surface effectively activated the phosphorylation of FAK at tyrosine 397 and β-catenin downstream factors in tumor cells. After inhibition of FAK phosphorylation, the proliferation and invasion ability of tumor cells were impaired, and influenced the nuclear accumulation of β-catenin and the EMT process (Fig. 5). Our data indicate that FAK signaling activation could be an important factor in the accumulation of nuclear β-catenin induced by matrix stiffness. However, further investigation is needed to determine the molecular mechanism by which the activation of the FAK pathway promotes OSCC progression. This is crucial for studying the occurrence and development of tumors.

## Conclusions

In summary, our results demonstrated that LOX expressed by CAFs could lead to the increase of matrix collagen stiffness and promote the progression of OSCC by activating FAK phosphorylation pathway. More experimental studies are needed to further reveal the detailed mechanisms underlying matrix stiffness and its cancer-promoting role in OSCC, which may be beneficial for the early diagnosis and prognosis of OSCC.

## Supplementary Information

**Additional file 1: Figure S1**. The expression patterns of α-SMA and LOX in tumor stroma. Representative images of high (A1-A3; B1-B3) and low (C1-C3; D1-D3) expression levels of α-SMA and LOX in tumor stroma are shown under different magnifications. LOX expression (B1-B3) was strongly positive in the same regions of α-SMA-positive CAFs (A1-A3). The same feature was shown in low expression regions (C1-C3; D1-D3). (A1-D1, 100 × ; A2-D2, 200 × ; A3-D3, 400 ×); Scale bar: 50 μm.

**Additional file 2: Figure S2.** Identification the characteristics of CAFs derived from human oral cancer tissues and loss-of-function assays. A, Representative images of human primary NFs and CAFs cells (100 ×). B, Gene expression of α-SMA, FSP-1 and FAP measured by RT-qPCR. C, Protein levels of α-SMA, FSP-1 and FAP determined by western blot. D, E, Efficient LOX knockdown by lentivirus transfection in CAFs was confirmed by RT-qPCR (D) and western blot assay (E). F, The migration ability in Cal27 and HN6 cells were evaluated by transwell assay. Representative images of migrated cells and quantification data were shown. G, Representative images of E-cadherin, vimentin expression in HN6 via immunofluorescence staining. Red: E-cadherin and vimentin; Blue: DAPI (400 ×). GAPDH served as loading control. β-actin served as loading control. The data are presented as the means ± SEM(n = 3); *P < 0.05, **P < 0.01, ***P < 0.001.

**Additional file 3: Fig. S3**. Matrix stiffness increased by drug modification promotes invasion ability of OSCC cells. A, Relative stiffness of collagen modified by huLOX, ribose and BAPN under stable condition was shown. Elastic modulus (G’) were measured by MARS60 microinfrared rheometer. B, Representative immunofluorescence images of F-actin staining in Cal27 cells were shown. Green: F-actin; Blue: DAPI (400 ×). C, The ability of Cal27 cells invaded collagen gels with different stiffness was determined by transwell assay. Representative images of invaded cells and quantification data were shown. D, The protein expression of EMT markers in Cal27 cells cultured on collagen gels were measured by western blot assays. β-actin served as loading control. The data are presented as the means ± SD; *P < 0.05, **P < 0.01, ***P < 0.001, ****P < 0.0001.

**Additional file 4: Fig. S4** Collagen of different stiffness activated the FAK and β-catenin signaling pathway in OSCC cells. A, The protein expression of p-FAK, FAK and β-catenin downstream proteins c-myc and cyclin D1 were measured by western blot assay in Cal27 cells cultured on collagen gels. B, The protein expression of p-FAK and FAK were shown after Cal27 cells treated with different concentrations of FAKi by western blot. C, The protein expression of β-catenin from the cytoplasmic and nuclear extracts were measured after the FAKi treatment by western blot. β-actin served as the cytoplasmic internal control. Lamin B served as the nuclear internal control. *P < 0.05, **P < 0.01, ***P < 0.001.

## Data Availability

All data generated or analyzed during this study are included in this published article and its Additional files.

## Abbreviations

CAFs: Cancer-associated fibroblasts
CM: Conditioned medium
ECM: Extracellular matrix
EMT: Epithelial–mesenchymal transition
FAK: Focal adhesion kinase
FAKi: FAK inhibitor 14
LOX: Lysyl oxidase
NFs: Normal fibroblasts
OSCC: Oral squamous cell carcinoma
TME: Tumor microenvironment
α-SMA: α-Smooth muscle actin
