# Supplementary material for: Cancer-associated fibroblasts promote oral squamous cell carcinoma progression through LOX-mediated matrix stiffness
Source: J Transl Med. 2021 Dec 20;19:513. doi: 10.1186/s12967-021-03181-x (PMC8686394; doi:10.1186/s12967-021-03181-x)
Supplement: Supplementary file 1 — Additional file 1: Figure S1. The expression patterns of α-SMA and LOX in tumor stroma. Representative images of high (A1-A3; B1-B3) and low (C1-C3; D1-D3) expression levels of α-SMA and LOX in tumor stroma are shown under different magnifications. LOX expression (B1-B3) was strongly positive in the same regions of α-SMA-positive CAFs (A1-A3). The same feature was shown in low expression regions (C1-C3; D1-D3). (A1-D1, 100 × ; A2-D2, 200 × ; A3-D3, 400 ×); Scale bar: 50 μm. [file 12967_2021_3181_MOESM1_ESM.docx]

**Additional file 1:**

**
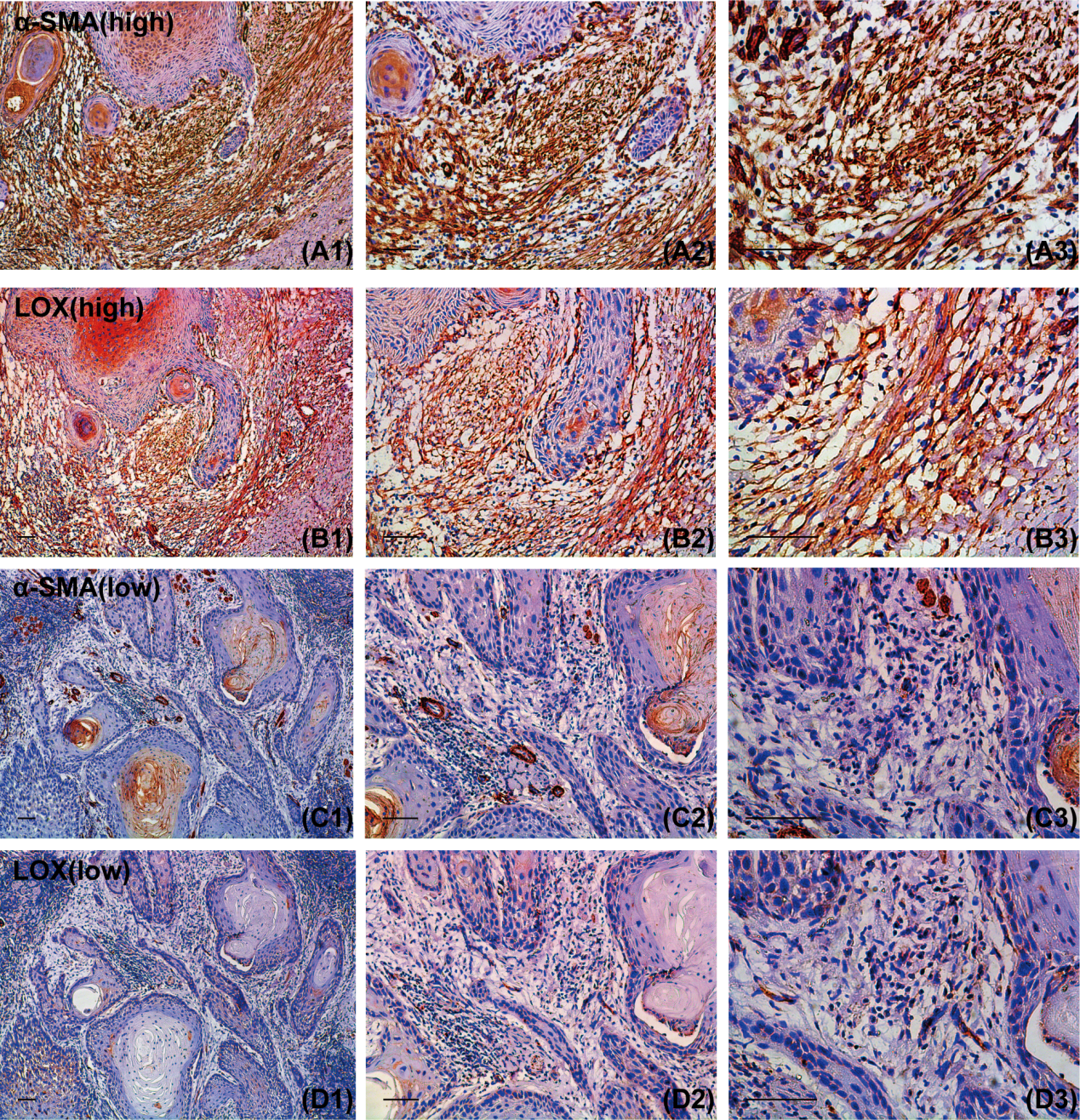
Figure S1** The expression patterns of α-SMA and LOX in tumor stroma. Representative images of high (A1-A3; B1-B3) and low (C1-C3; D1-D3) expression levels of α-SMA and LOX in tumor stroma are shown under different magnifications. LOX expression (B1-B3) was strongly positive in the same regions of α-SMA-positive CAFs (A1-A3). The same feature was shown in low expression regions (C1-C3; D1-D3). (A1-D1, 100×; A2-D2, 200×; A3-D3, 400×); Scale bar: 50μm.
