# Supplementary material for: Cancer-associated fibroblasts promote oral squamous cell carcinoma progression through LOX-mediated matrix stiffness
Source: J Transl Med. 2021 Dec 20;19:513. doi: 10.1186/s12967-021-03181-x (PMC8686394; doi:10.1186/s12967-021-03181-x)
Supplement: Supplementary file 2 — Additional file 2: Figure S2. Identification the characteristics of CAFs derived from human oral cancer tissues and loss-of-function assays. A, Representative images of human primary NFs and CAFs cells (100 ×). B, Gene expression of α-SMA, FSP-1 and FAP measured by RT-qPCR. C, Protein levels of α-SMA, FSP-1 and FAP determined by western blot. D, E, Efficient LOX knockdown by lentivirus transfection in CAFs was confirmed by RT-qPCR (D) and western blot assay (E). F, The migration ability in Cal27 and HN6 cells were evaluated by transwell assay. Representative images of migrated cells and quantification data were shown. G, Representative images of E-cadherin, vimentin expression in HN6 via immunofluorescence staining. Red: E-cadherin and vimentin; Blue: DAPI (400 ×). GAPDH served as loading control. β-actin served as loading control. The data are presented as the means ± SEM(n = 3); *P < 0.05, **P < 0.01, ***P < 0.001. [file 12967_2021_3181_MOESM2_ESM.docx]

**Additional file 2:**

**
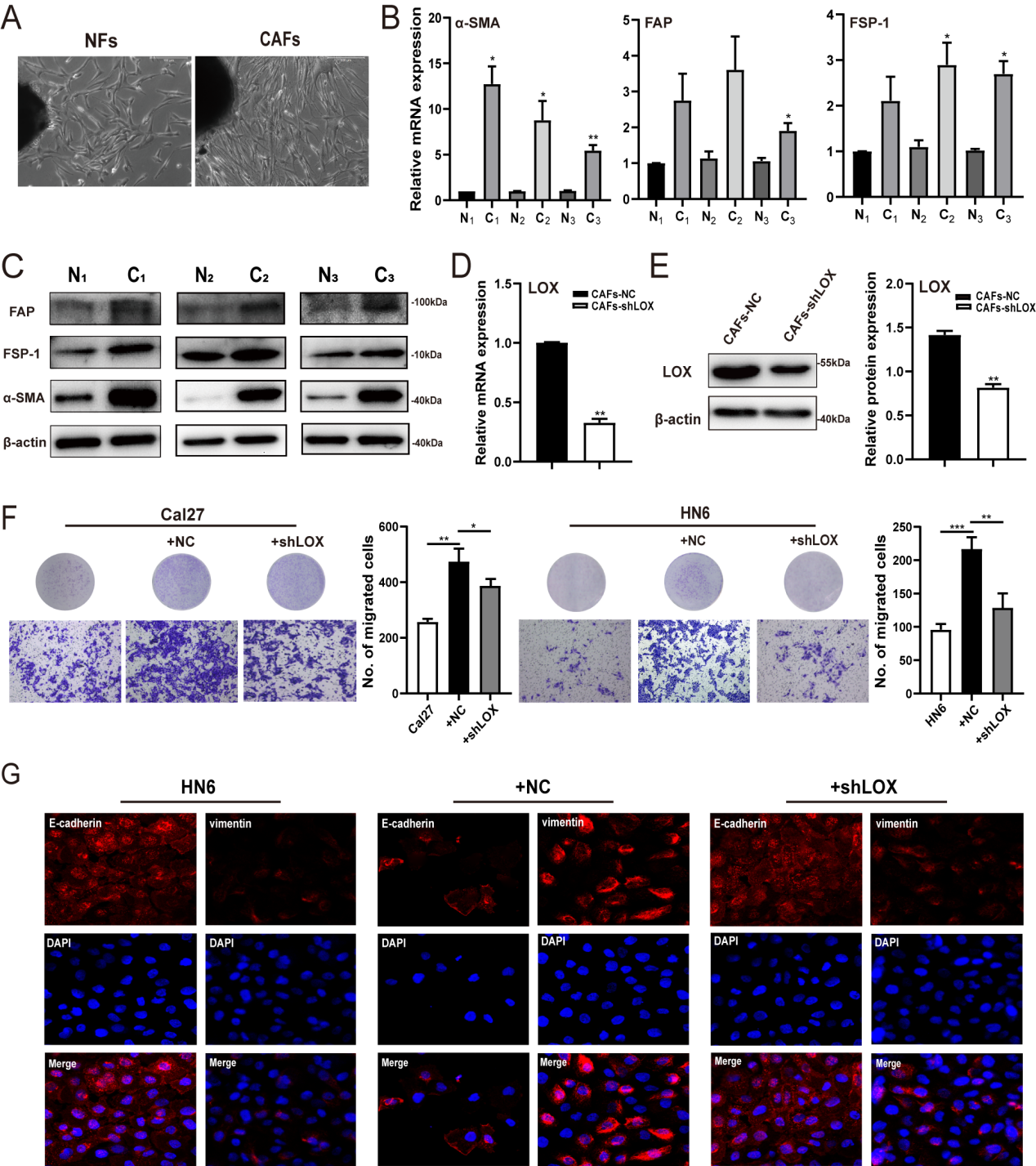
**

**Figure S2** Identification the characteristics of CAFs derived from human oral cancer tissues and loss-of-function assays. A, Representative images of human primary NFs and CAFs cells (100×). B, Gene expression of α-SMA, FSP-1 and FAP measured by RT-qPCR. C, Protein levels of α-SMA, FSP-1 and FAP determined by western blot. D, E, Efficient LOX knockdown by lentivirus transfection in CAFs was confirmed by RT-qPCR (D) and western blot assay (E). F, The migration ability in Cal27 and HN6 cells were evaluated by transwell assay. Representative images of migrated cells and quantification data were shown. G, Representative images of E-cadherin, vimentin expression in HN6 via immunofluorescence staining. Red: E-cadherin and vimentin; Blue: DAPI (400×). GAPDH served as loading control. β-actin served as loading control. The data are presented as the means ± SEM(n=3); *P < 0.05, **P < 0.01, ***P < 0.001.
