# Supplementary material for: Cancer-associated fibroblasts promote oral squamous cell carcinoma progression through LOX-mediated matrix stiffness
Source: J Transl Med. 2021 Dec 20;19:513. doi: 10.1186/s12967-021-03181-x (PMC8686394; doi:10.1186/s12967-021-03181-x)
Supplement: Supplementary file 3 — Additional file 3: Fig. S3. Matrix stiffness increased by drug modification promotes invasion ability of OSCC cells. A, Relative stiffness of collagen modified by huLOX, ribose and BAPN under stable condition was shown. Elastic modulus (G’) were measured by MARS60 microinfrared rheometer. B, Representative immunofluorescence images of F-actin staining in Cal27 cells were shown. Green: F-actin; Blue: DAPI (400 ×). C, The ability of Cal27 cells invaded collagen gels with different stiffness was determined by transwell assay. Representative images of invaded cells and quantification data were shown. D, The protein expression of EMT markers in Cal27 cells cultured on collagen gels were measured by western blot assays. β-actin served as loading control. The data are presented as the means ± SD; *P < 0.05, **P < 0.01, ***P < 0.001, ****P < 0.0001. [file 12967_2021_3181_MOESM3_ESM.docx]

**Additional file 3:
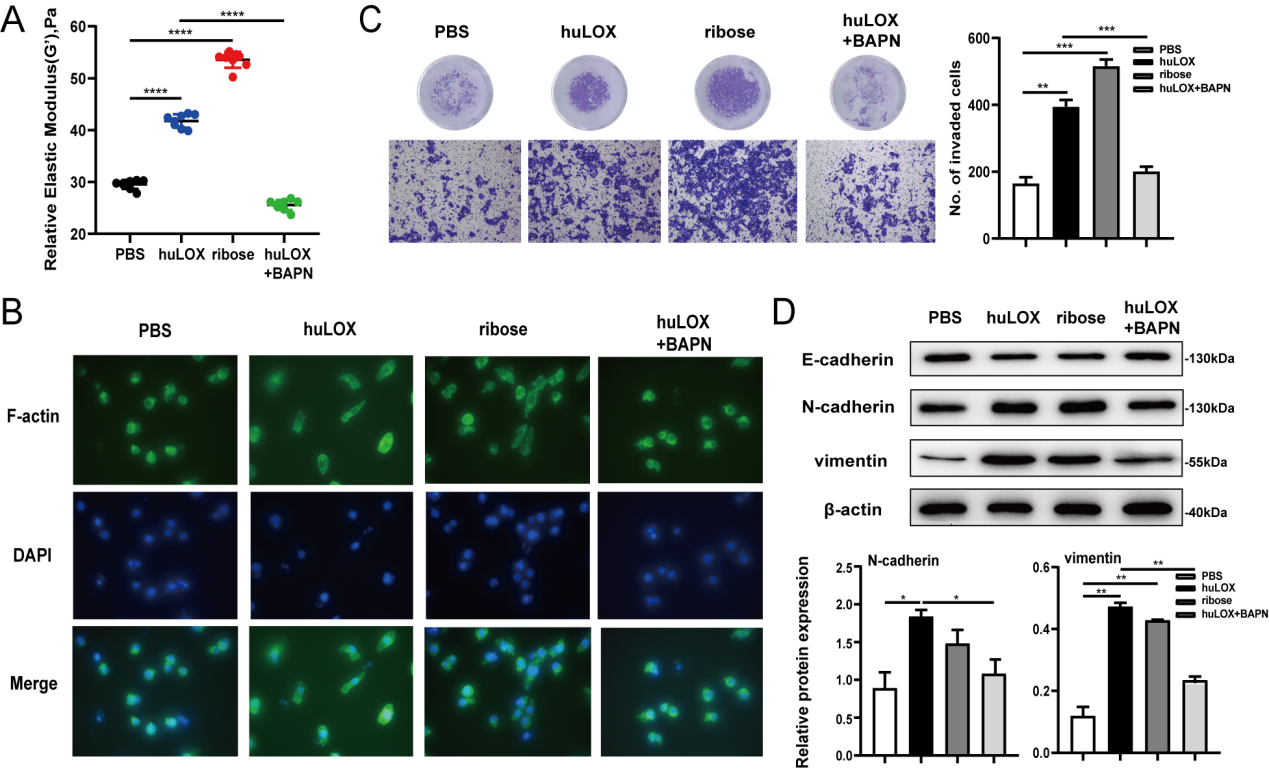
**

**Figure S3** Matrix stiffness increased by drug modification promotes invasion ability of OSCC cells. A, Relative stiffness of collagen modified by huLOX, ribose and BAPN under stable condition was shown. Elastic modulus (G’) were measured by MARS60 microinfrared rheometer. B, Representative immunofluorescence images of F-actin staining in Cal27 cells were shown. Green: F-actin; Blue: DAPI (400×). C, The ability of Cal27 cells invaded collagen gels with different stiffness was determined by transwell assay. Representative images of invaded cells and quantification data were shown. D, The protein expression of EMT markers in Cal27 cells cultured on collagen gels were measured by western blot assays. β-actin served as loading control. The data are presented as the means ± SD; *P < 0.05, **P < 0.01, ***P < 0.001, ****P < 0.0001.
