# Supplementary material for: Cancer-associated fibroblasts promote oral squamous cell carcinoma progression through LOX-mediated matrix stiffness
Source: J Transl Med. 2021 Dec 20;19:513. doi: 10.1186/s12967-021-03181-x (PMC8686394; doi:10.1186/s12967-021-03181-x)
Supplement: Supplementary file 4 — Additional file 4: Fig. S4 Collagen of different stiffness activated the FAK and β-catenin signaling pathway in OSCC cells. A, The protein expression of p-FAK, FAK and β-catenin downstream proteins c-myc and cyclin D1 were measured by western blot assay in Cal27 cells cultured on collagen gels. B, The protein expression of p-FAK and FAK were shown after Cal27 cells treated with different concentrations of FAKi by western blot. C, The protein expression of β-catenin from the cytoplasmic and nuclear extracts were measured after the FAKi treatment by western blot. β-actin served as the cytoplasmic internal control. Lamin B served as the nuclear internal control. *P < 0.05, **P < 0.01, ***P < 0.001. [file 12967_2021_3181_MOESM4_ESM.docx]

**Additional file 4:**

**
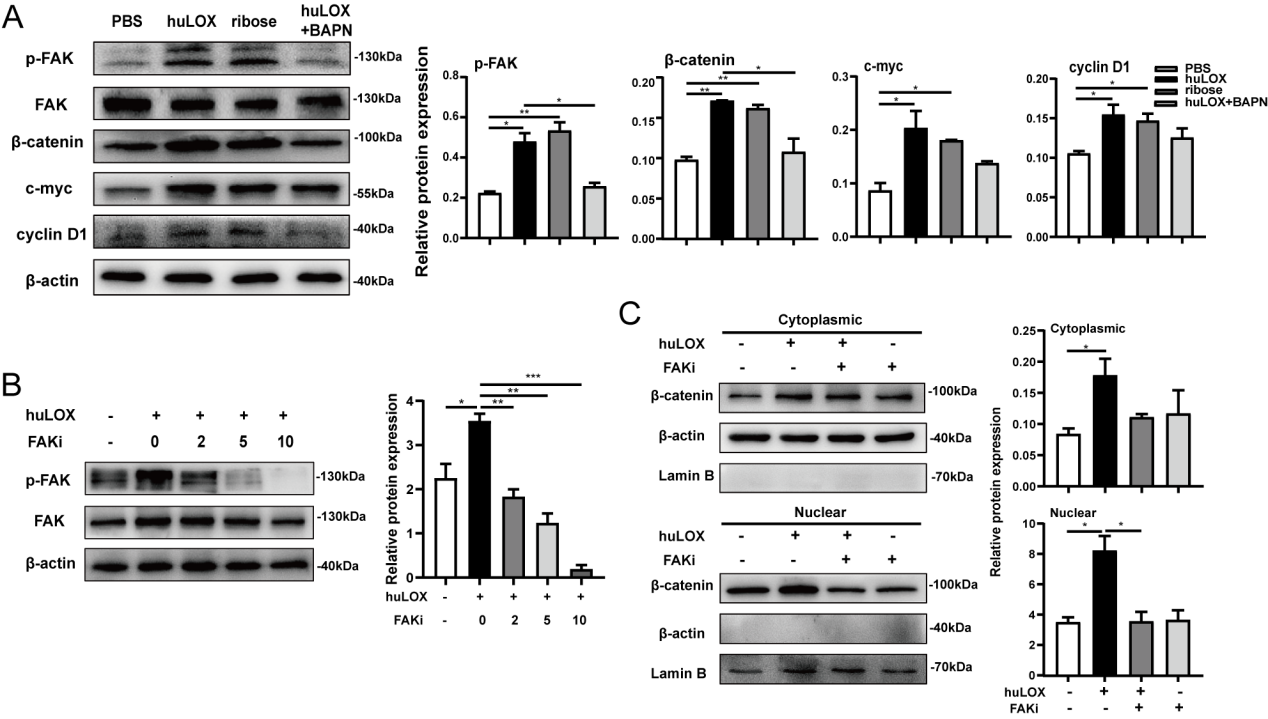
**

**Figure S4** Collagen of different stiffness activated the FAK and β-catenin signaling pathway in OSCC cells. A, The protein expression of p-FAK, FAK and β-catenin downstream proteins c-myc and cyclin D1 were measured by western blot assay in Cal27 cells cultured on collagen gels. B, The protein expression of p-FAK and FAK were shown after Cal27 cells treated with different concentrations of FAKi by western blot. C, The protein expression of β-catenin from the cytoplasmic and nuclear extracts were measured after the FAKi treatment by western blot. β-actin served as the cytoplasmic internal control. Lamin B served as the nuclear internal control. *P < 0.05, **P < 0.01, ***P < 0.001.
